# Accuracy of Ultrasound and Computed Tomography in Diagnosing Acute Cholecystitis Patients in a Tertiary Care Center in Saudi Arabia

**DOI:** 10.7759/cureus.44934

**Published:** 2023-09-09

**Authors:** Mawya A Khafaji, Juman T Bagasi, Sarah K Albahiti, Lama A Alsayegh, Shahd A Alsayyad, Seba S Algarni, Sarah Y Bahowarth, Ebtihal S Baghdadi

**Affiliations:** 1 Radiology Department, Faculty of Medicine, King Abdulaziz University Hospital, Jeddah, SAU; 2 Faculty of Medicine, King Abdulaziz University Hospital, Jeddah, SAU

**Keywords:** negative predictive value (npv), positive predictive value (ppv), specificity, sensitivity, accuracy, ct, computed tomography, us, ultrasound, acute cholecystitis

## Abstract

Background

Acute cholecystitis (AC) is a gallbladder inflammatory disease often associated with gallbladder stones. It accounts for up to 5% of emergency department visits. The majority of patients present with pain in the right upper quadrant, Murphy’s sign, and fever. Furthermore, Saudi Arabia has been noted to have a significant prevalence of AC. According to the 2018 Tokyo Guidelines, imaging is an essential element, combined with local and systemic evidence of inflammation, for a confirmed diagnosis of AC. The definitive therapy is conducted surgically by cholecystectomy either urgently or electively. However, there are insufficient studies that focus on the accuracy of imaging in diagnosing AC patients in Saudi Arabia.

Objective

The aim of this study is to assess the accuracy of ultrasound (US) versus computed tomography (CT) in diagnosing AC patients at King Abdulaziz University Hospital (KAUH), Jeddah, Saudi Arabia.

Methods and material

A retrospective record review was conducted at KAUH during the period of June to July 2022. The study included 192 patients diagnosed with AC in the emergency department or outpatient department by US or CT or both and confirmed by laparoscopic cholecystectomy and histopathology between 2016 and 2022.

Results

The most common modality used was US (79.7%), followed by both US and contrast CT (10.9%). For CT, sensitivity was 81.3%, specificity was 62.5%, positive predictive value (PPV) was 59.1%, and negative predictive value (NPV) was 83.3%. For US, sensitivity was 37.9%, specificity was 81.7%, PPV was 50%, and NPV was 73.1%. A significant relationship was observed between both genders and high use of US (P = 0.0001).

Conclusion

We found that CT is more sensitive than US, while US is more specific in diagnosing AC.

## Introduction

Acute cholecystitis (AC) is an inflammatory disease of the gallbladder that is often associated with gallstones [1]. Saudi Arabia has been noted to have a significant prevalence of AC of around 24% and a female-to-male ratio of 11.9:1, while the incidence of AC was 4.4/per 100,000 individuals per year [2]. Aside from being encountered in surgical practice, it accounts for up to 5% of emergency department (ED) visits [3,4]. Most patients present with right upper quadrant (RUQ) pain and fever [5]. Additionally, in 18.5% of AC cases, complicated intra-abdominal infection is the considered as the second most cause of AC [6]. Risk factors for AC include female sex, obesity, pregnancy, and those in their forties [1]. Also, it affects about one-third of adults with cholelithiasis [7]. Each year, 1-4% of cholelithiasis patients appear with symptoms, and some of these (30%) develop AC [8-10]. The favored initial imaging test for the diagnosis of AC is ultrasound (US), while the preferable alternative is scintigraphy. Computed tomography (CT) is a secondary imaging examination that can detect extrabiliary conditions and AC complications [11]. US utilizes high-frequency sound waves to produce images of internal organs with no radiation exposure, and it normally takes 10 to 15 minutes to complete. On the other hand, CT produces a cross-sectional image of the body by using computers and rotating X-ray devices. Typically, CT scans last 10 to 30 minutes. The radiation dose ranges from 2 to 10 mSv; therefore, it is not recommended in children and pregnant women [12]. The definitive therapy is conducted surgically by cholecystectomy either urgently or electively [13].

A cross-sectional study conducted in 2018 in Saudi Arabia at King Faisal Medical Complex found that the majority of patients diagnosed with chronic cholecystitis presented to the outpatient department ([OPD] 57%), while those diagnosed with AC presented to the ED (43%) [14]. A previous study conducted in the United States of America in 2003 confirmed that medical history and physical examination findings cannot detect AC in the clinical setting [10]. On the contrary, a study in 2017 confirmed that AC could be diagnosed based on a patient's medical history, physical examination, laboratory tests, and US [15]. Despite the availability of various imaging modalities for diagnosing AC, each has a distinct role to play in the condition's management, and sensitivity, specificity, and diagnostic accuracy values vary significantly [15]. Accordingly, imaging is critical in the workup of these patients because it speeds up the diagnosis process and may reveal complications such as gangrene and perforation [16-18]. As a result, the 2018 Tokyo Guidelines retained imaging as a necessary component of a definitive diagnosis of AC, along with local and systemic signs of inflammation [19]. US has long been considered as the gold standard for cholecystitis imaging diagnosis due to its wide availability, lack of ionizing radiation, quick acquisition of images, and reasonable cost. However, the use of CT as the primary imaging modality for patients experiencing abdominal pain has increased with improvements in technology and availability [20,21]. Nevertheless, in Singapore, a study reported that CT has a low sensitivity for detecting gallstones due to the varied composition of the stones and that even larger isodense gallstones could not show up on a CT scan [22].

In a recent retrospective study conducted in France, CT scans and US were found equally effective in identifying AC. However, a CT scan is more effective in diagnosing complicated cases. Therefore, US had a higher sensitivity than the CT scan but a lower specificity [6]. In addition, multiple studies found that CT is superior to US in diagnosing cholecystitis in adult patients undergoing emergency evaluation for RUQ pain and has the advantage of depicting acute non-gallbladder abnormalities [23-25]. Another study reported that US in pediatric patients with cholecystitis is less sensitive than in adults [21]. Furthermore, Saudi Arabia has been noted to have a significant prevalence of AC. However, studies on the accuracy of imaging in diagnosing AC are insufficient. Therefore, this study aims to assess the accuracy of US versus CT in diagnosing AC in patients admitted to King Abdulaziz University Hospital (KAUH), Jeddah, Saudi Arabia, between the years 2016 and 2022.

## Materials and methods

This retrospective record review study was conducted at KAUH, a tertiary care center in Jeddah, Saudi Arabia, between June and July 2022. This research was approved by the Institutional Review Board of KAUH (Ref: 78-22). Due to the study's retrospective nature, informed consent was waived.

Initially, data from 559 patients were collected. Patients who were confirmed to have AC by laparoscopic cholecystectomy (regardless of the route of admission) and who underwent CT or US or both between January 1, 2016, and January 1, 2022, were included. Out of 559 patients, 192 met the inclusion criteria. Patients with no radiological investigation, pregnant women, patients who were transferred from other hospitals were excluded.

Clinical data

The data of all patients diagnosed with AC were acquired through a review of the hospital's electronic records. Data retrieved included information about the route of admission (ED or OPD), type of shift if the patient was admitted through ED, height (cm), weight (kg), first clinical presentation including RUQ pain, epigastric pain, unspecified abdominal pain, fever, pain radiated to the shoulder or back, nausea, and vomiting. Other concomitant morbidities were also noted Laboratory values included in the study were total white blood cell (WBC) count, aspartate transaminase (AST), alanine transaminase (ALT), total and direct bilirubin, and gamma-glutamyltransferase (GGT).

Radiological data

US findings were based on the final attending radiologist's interpretation and included the presence of a sonographic Murphy's sign, gallbladder wall thickening (defined as a gallbladder wall >3 mm), pericholecystic fluid, gallstones, gallbladder sludge, enlarged/distended gallbladder, common bile duct dilatation (>6 mm), and air within the gallbladder lumen or wall (dirty shadowing). Patients were considered positive for AC if they had any of the previously mentioned findings. For each CT study, the following findings were documented: presence of IV contrast material, gallstones, pericholecystic fluid, pericholecystic inflammation, common bile duct dilatation (defined as >6 mm), increased enhancement of the adjacent liver, gallbladder wall thickening (defined as >3 mm), indistinct gallbladder wall, choledocholithiasis, air within the gallbladder lumen or wall, increased gallbladder wall attenuation, and poor gallbladder wall. A positive diagnosis of AC was determined based on the presence of at least two of the previously described findings, although no specific set of findings was considered to make the diagnosis of AC.

Pathological data

Pathological findings were interpreted by pathologists assigned to each case. Characteristics present in specimens interpreted as AC included edema, hemorrhage, mucosal ulceration, fibrin deposition, neutrophilic infiltrate, and necrosis. Chronic cholecystitis specimens included mononuclear infiltration, papillary mucosa lined by mucin-secreting cells, Rokitansky-Aschoff sinuses in the wall, fibromuscular hyperplasia, and lipid-laden macrophages in the lamina propria.

Statistical analysis

Data were entered into Microsoft Excel and the Statistical Package for Social Sciences (SPSS) Version 25 (IBM Corp., Armonk, NY, USA) was used for statistical analysis. GraphPad Prism Version 5 (GraphPad Software, Inc., San Diego, CA, USA) was used to transmit information visually. Sensitivity, specificity, negative predictive value (NPV), and positive predictive value (PPV) for US and CT were calculated. Means±standard deviations (SDs) were determined to describe continuous variables, while frequencies and percentages were used for categorical variables. The chi-square test and one-way ANOVA test were used to compare between categorical and continuous variables, respectively. Statistical significance was considered at (P < 0.05).

## Results

A total of 192 patients were included in the analysis. Patients' ages ranged from 4 to 80 years, with a mean±SD age of 42.4±15.6 years. Additionally, the mean± SD body mass index was 29.13±7.05 kg/m^2^. Females were more prevalent than males (63.5% versus 36.5%). Diabetes mellitus (15.6%) and hypertension (15.6%) constituted the majority of the comorbid diseases.

Most of the patients were admitted through ED (70.3%). The most presenting manifestations were RUQ pain (58.9%), vomiting (49.3%), and unspecified abdominal pain (40.1%). The most common radiological modality used was US (79.7%), followed by both US and contrast CT (10.9%) (Table 1).

**Table 1:** Demographic and clinical characteristics of the studied patients (n= 192) Note: Categorical variables are expressed as frequency or count (%) and continuous variables as mean±SD. SCA, sickle cell anemia; G6PD, glucose-6-phosphate dehydrogenase deficiency; ED, emergency department; OPD, outpatient department; RUQ, right upper quadrant; US, ultrasound; CT, computed tomography; SD, standard deviation

Variables	Value
Age (years)	42.41±15.63 (4-80)
Weight (kg)	75.94±20.19 (12-132)
Height (cm)	160.85±12.44 (100-193)
Body mass index (kg/m^2^)	29.13±7.05 (11.50-54.53)
Gender	
Male	70 (36.5%)
Female	122 (63.5%)
Comorbidities	
Diabetes mellitus	30 (15.6%)
Hypertension	30 (15.6%)
Dyslipidemia or hyperlipidemia or abnormal lipid profile	10 (5.2%)
Thyroid disease	9 (4.7%)
Ischemic heart disease	8 (4.2%)
SCA	7 (3.6%)
Chronic heart disease	4 (2.1%)
Neurological disease	4 (2.1%)
Psychiatric disorder	4 (2.1%)
Chronic liver disease	3 (1.6%)
Respiratory disease	3 (1.6%)
G6PD deficiency	2 (1.0%)
Spherocytosis	2 (1.0%)
Rheumatological disease	1 (0.5%)
Malignancy	1 (0.5%)
Patient was admitted through	
ED	135 (70.3%)
OPD	57 (29.7%)
Manifestations	
RUQ pain	113 (58.9%)
Vomiting	95 (49.3%)
Unspecified abdominal pain	77 (40.1%)
Nausea	58 (30.2%)
Pain radiated to the shoulder and back	58 (30.2%)
Epigastric pain	48 (25.0%)
Fever	23 (12.0%)
Radiological modality used	
US	153 (79.7%)
Contrast CT	8 (4.2%)
Non-contrast CT	6 (3.1%)
Both US and contrast CT	21 (10.9%)
US and non-contrast CT	4 (2.1%)

**Figure 1:**
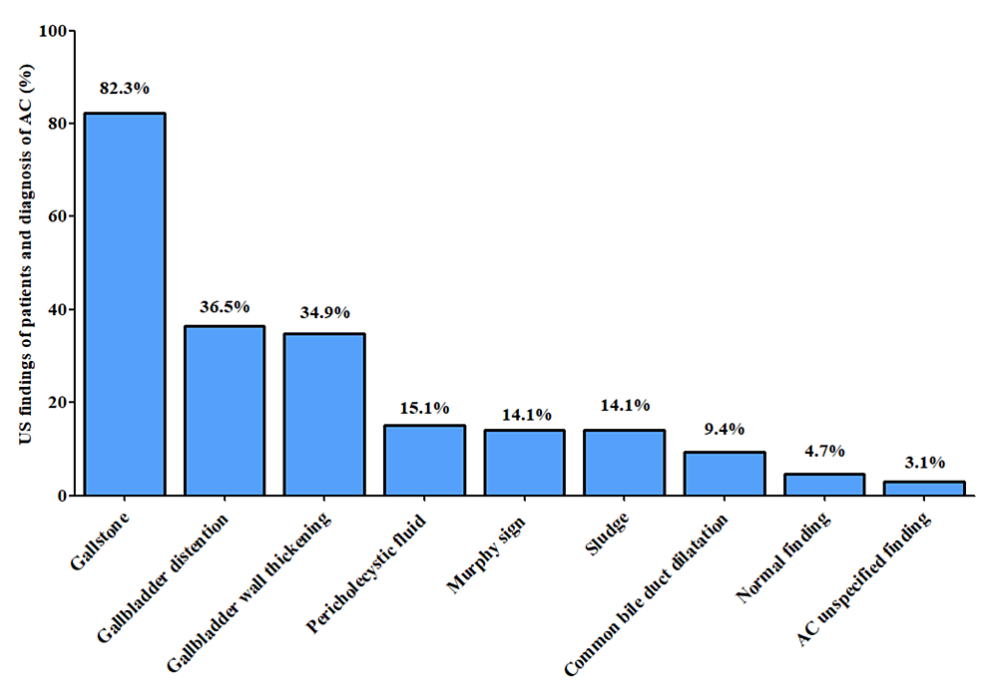
Ultrasound findings in patients diagnosed with acute cholecystitis (n=178)

As shown in Figure 1, 178 patients were diagnosed by US, and the most common US findings were gallstones (82.3%), gallbladder distention (> 4 cm) (36.5%), and gallbladder wall thickening (>3 mm) (34.9%).

**Figure 2:**
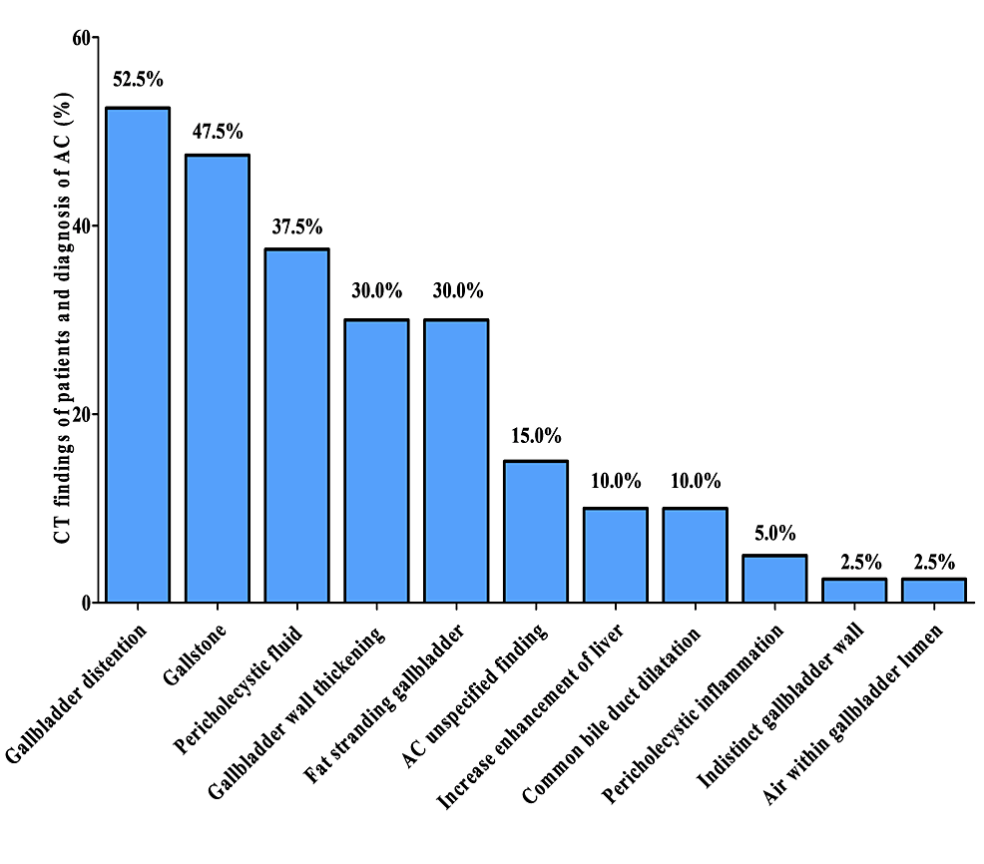
Computed tomography findings in patients diagnosed with acute cholecystitis (n=40)

As shown in Figure 2, among 40 patients diagnosed by CT, the most common CT findings were gallbladder distention (> 4 cm) (52.5%), gallstone (47.5%), and pericholecystic fluid (37.5%).

The WBC count and results of the liver function tests are shown in Table 2. WBC count > 11,000/µL was reported in 49 (25.5%) cases, AST > 300 IU/L in nine (4.7%) cases, ALT > 50 IU/L in 58 (30.2%) cases, and GGT > 168 IU/L in 38 (19.8%) cases.

**Table 2:** Laboratory findings of patients WBC, white blood cell; AST, aspartate transaminase; ALT, alanine transaminase; GGT, gamma-glutamyltransferase

Variable	Values
Total WBC count	9.05±4.49 (2.58-38.38)
AST	66.26±133.33 (8 -1188)
ALT	72.83±117.06 (7-959)
GGT	107.62±168.27 (3-988)
Total bilirubin (mg/dL)	15.67±19.37 (2.40-163)
Direct bilirubin (mg/dL)	13.20±15.22 (2-62)
WBC count > 11,000/µL	49 (25.5%)
ALT > 50 IU/L	58 (30.2%)
AST > 300 IU/L	9 (4.7%)
GGT > 168 IU/L	38 (19.8%)

**Figure 3:**
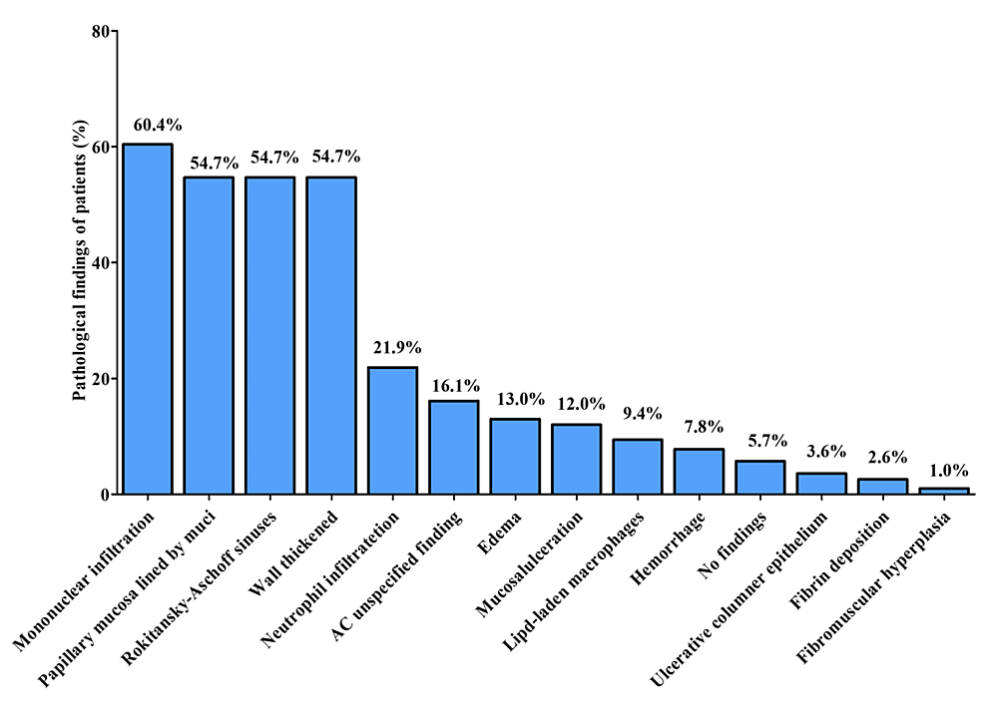
Pathological findings of gallbladder after cholecystectomy (n=192)

After surgical excision of the gall bladder, histopathological examination of the gall bladder revealed mononuclear infiltration in 116 (60.4%) cases, papillary mucosa lined by mucin-secreting cells in 105 (54.7%) cases, Rokitansky-Aschoff sinuses in the wall in 105 (54.7%) cases, and wall thickening in 105 (54.7%) cases (Figure 3).

**Figure 4:**
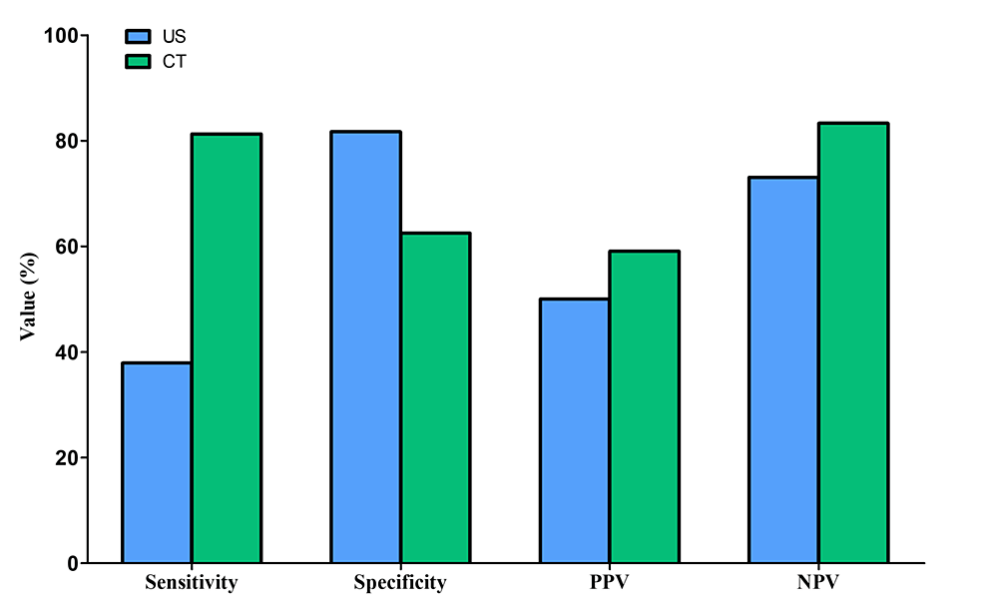
Sensitivity, specificity, NPV, and PPV for US and CT in patients diagnosed with acute cholecystitis NPV, negative predictive value; PPV, positive predictive value; US, ultrasound; CT, computed tomography

Out of 44 cases that were diagnosed as AC by US imaging, 22 cases were correctly identified as AC (true positive) and 22 were incorrectly diagnosed as AC (false positive). While 134 cases were diagnosed as negative for AC, 98 of them were true negative and 36 were false negative. From these results, for US, sensitivity was 37.9%, specificity was 81.7%, PPV was 50%, and NPV was 73.1%. Using CT, 22 cases were diagnosed as AC. Of those, 13 cases were correctly identified as AC (true positives), and nine cases were incorrectly diagnosed as AC (false positives). While 18 cases were diagnosed as negative for AC, of those, 15 cases were true negative and three cases were false negative. According to these findings, for CT, sensitivity was 81.3%, specificity was 62.5%, PPV was 59.1%, and NPV was 83.3% (Table 3 and Figure 4).

**Table 3:** Imaging status of patients US, ultrasound; AC, acute cholecystitis; CT, computed tomography

Diagnosis by US	Values
Diagnosed as AC by US	44 (24.7%)
True positive	22 cases
False negative	36 cases
True negative	98 cases
False positive	22 cases
Diagnosis by CT	
Diagnosis as AC by CT	22 (55.0%)
True positive	13 cases
False negative	3 cases
True negative	15 cases
False positive	9 cases

Table 4 and Table 5 show imaging modalities used with age and sex. Out of 70 male patients, US was used in 48 (68.5%), while out of 122 female patients, US was used in 105 (86.1%) cases. Patients who underwent contrast CT were older than those who used other imaging modalities; however, these differences did not reach statistical significance (P = 0.514).

**Table 4:** Cross-tabulation between gender and imaging modality used US, ultrasound; CT, computed tomography

Variables	Male (n=70)	Female (n=122)	P-value
US (n = 153)	48 (68.5%)	105 (86.1%)	0.0001
Contrast CT (n = 8)	7 (10.0%)	1 (0.8%)	0. 314
Non-contrast CT (n = 6)	2 (2.9%)	4 (3.3%)	0.414
US and contrast CT (n = 21)	12 (17.1%)	9 (7.4%)	0.513
US and non-contrast CT (n = 4)	1 (1.4%)	3 (2.5%)	0.317

**Table 5:** Cross-tabulation between age and modality used US, ultrasound; CT, computed tomography

Variables	Age
US (n = 153)	41.68±15.50
Contrast CT (n = 8)	48.13±12.85
Non-contrast CT (n = 6)	40.67±14.05
US and contrast CT (n = 21)	46.71±18.27
US and non-contrast CT (n = 4)	38.75±12.82
P-value	0.514

**Figure 5:**
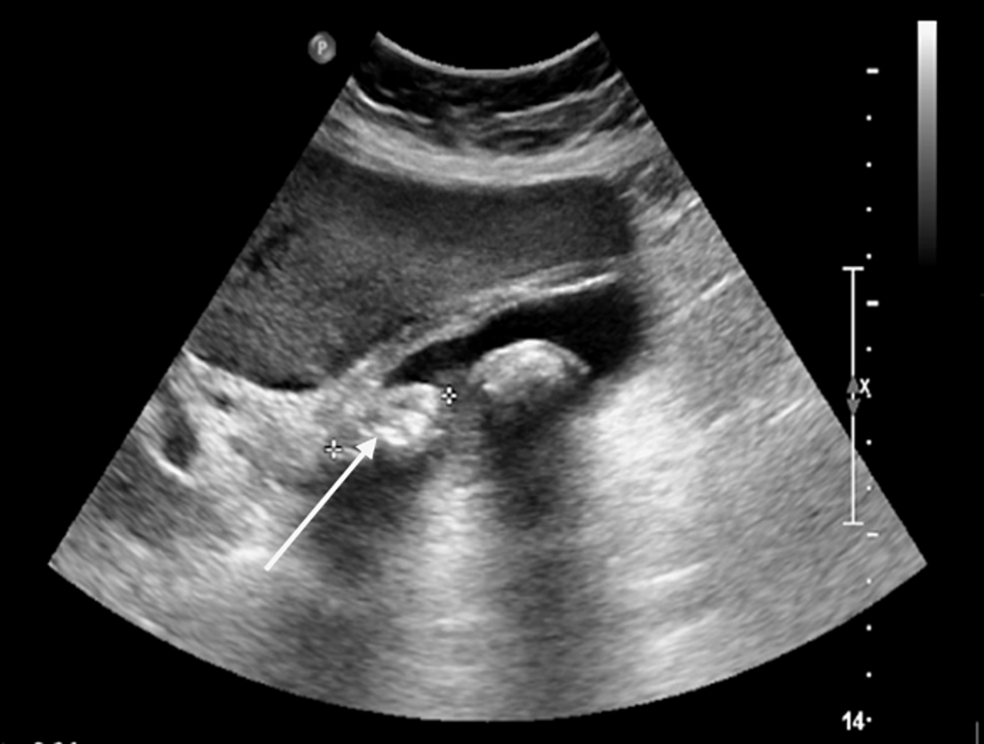
Ultrasound image shows gallstone, gallbladder thickening, pericholecystic fluid, and sonographic Murphy’s sign Arrow indicates gallstone

**Figure 6:**
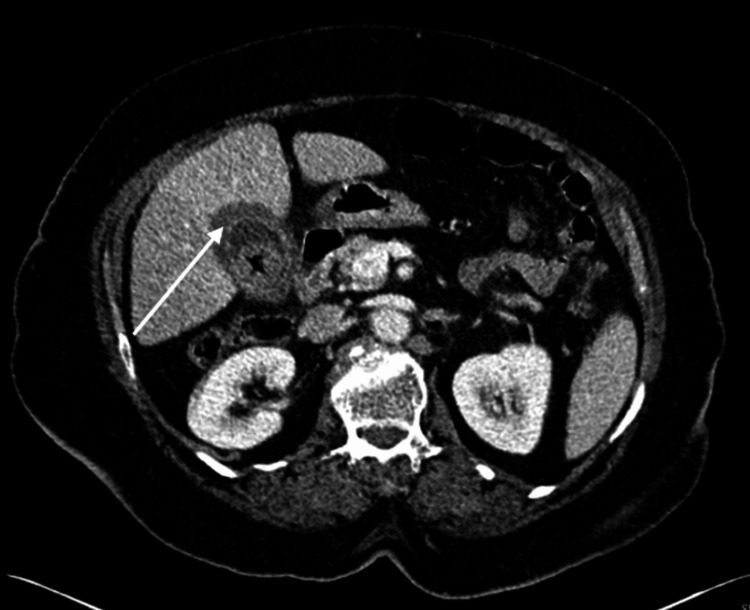
Computed tomography image shows gallstone, gallbladder thickening, and pericholecystic fluid Arrow indicates gallbladder thickening

Figure 5 and Figure 6 show the imaging of AC in an 80-year-female patient who was a known case of diabetes mellitus who presented to ED with RUQ pain radiating to the shoulder, fever, and nausea and vomiting.

## Discussion

Accuracy of US and CT

In this study, the CT sensitivity was found to be high in comparison to US (81.3% versus 37.9%). This finding appears to be similar to a previous study that found that the sensitivity of CT was significantly greater than US for detecting AC (85% versus 68%) [26]. When compared to the recent literature, Oda et al. reported that CT had more sensitivity than US (85% versus 72%) [24]. Additionally, consistent with our results, Compared to US, CT was more sensitive (79% versus 92%) [27]. This finding is contrary to previous studies that have suggested that US was more sensitive than the CT (61% versus 55%) [23]. A possible explanation for this might be that US examination is often performed by a technician with more experience, but during night shifts in the ED, weekends, and holidays, technicians may not always be accessible, resulting in a large number of examinations being performed by ED physicians, and this factor may contribute to CT's significantly higher sensitivity compared to US. This variation due to the different CT protocols used at various centers may have played a role in decreasing the sensitivity of CT for AC in their research.

US most used in both gender

According to our findings, a significant relationship was observed between both genders and high use of US (P = 0.0001). However, we observed that females were diagnosed by US more than males (86.1% versus 68.5%). A possible explanation for the widespread use of US technology in females is that it can create images of internal organs without the need for radiation. Furthermore, it can examine a female's fallopian tubes, uterus, vagina, cervix, and endometrium in order to identify any pathology. It is also used for pregnancy or in suspected pregnancy due to its safety. A consistent study found that US was the most common single imaging study performed for AC [23]. Furthermore, multiple studies showed that US is the best imaging method and the gold standard for assessing AC; signs and symptoms are frequently used to diagnose AC [17,19,28]. Another study conducted in Boston by Fagenholz et al. reported that in comparison to individuals who underwent both CT and US, patients who underwent US alone were younger and had fewer medical comorbidities. They were also much more likely to show classic signs of AC, such as RUQ pain or Murphy’s sign [27]. Due to its lower relative cost, broad availability, fast acquisition of the image, lack of ionizing radiation or contrast material, superior ability to detect gallstones, and addition of the sonographic Murphy’s sign, US has been favored over CT in the diagnosis of AC [29]. However, US is also limited in its ability to detect other intra-abdominal pathology if there are no specific gallstone symptoms. In addition, cholescintigraphy (hepatobiliary iminodiacetic acid [HIDA] scan) has been recommended in patients with suspected AC where US and clinical indicators have not proven the diagnosis. Although being both sensitive and specific, its cost is comparable to that of CT. It also utilizes ionizing radiation, and it shares some of US's limitations such as the inability to assess alternative diagnoses [27]. Therefore, this suggests that a diagnostic assessment that begins with US may need additional diagnostic tests.

US findings

In our institution, it was found that the most common US finding is gallstones. Similarly, a previous study in Romania showed that US is highly accurate in diagnosing cholelithiasis [30]. Several studies also noted that US is an efficient imaging modality in diagnosing gallbladder stones [16,17,31]. Therefore, we believe that this similarity is due to the machine’s unique nature in delivering the sound waves into the body so that the stone reflects these waves, causing it to appear bright. Additionally, these waves cannot penetrate the stone, which generates a shadow behind the stone, leading it to appear even more.

CT findings

Regarding the CT findings, we found that gallbladder distension > 4 cm is the most prevalent finding. This is probably caused by the gallbladder enlarging because of the cystic duct becoming blocked, which leads to a buildup of sterile, non-pigmented mucin. A study conducted by Fidler et al. in America reported that AC patients were more likely to develop gallbladder distension, although this is a nonspecific finding for AC [32]. Contrary to this finding, a study conducted in New York found that gallbladder distension and AC did not correlate well [33]. However, we believe that CT allows better evaluation of pathologies.

Contrast CT and elderly patients

Unexpected finding suggests that CT with contrast is predominantly used in the older patients in our study. Fagenholz et al. demonstrated that the presence of cholelithiasis on US combined with a typical clinical picture often provides sufficient diagnostic accuracy in younger, healthier patients [26]. Meanwhile, multiple other studies suggest that CT imaging has better sensitivity and accuracy in diagnosing AC, especially when there is an atypical presentation and comorbidities [16,26,27]. As our results demonstrated, a large percentage of our patients have comorbidities, and older patients more typically suffer from comorbidities, which may explain the need for contrast CT for older patients. Regarding sensitivity, contrast CT helps in revealing the gallbladder and adjacent liver tissue. However, contrast material is a risk factor for acute renal injury in the elderly with comorbidities, and we believe that this is a relevant issue for further research in our hospital.

Limitations

Our study includes limitations that must be addressed in future research, such as a small sample size. Thus, a larger sample size is required to have a more accurate calculation for sensitivity, specificity, PPV, and NPV, and to compare the accuracy between adults and pediatrics. Additionally, poor radiological and pathological documentations are limited to one center only. Therefore, a multicenter study is recommended to compare accuracies between different centers.

## Conclusions

The current study's main goal was to determine US and CT's sensitivity and specificity in predicting AC in relation to histopathology reports. We found that CT was more sensitive than US, while US is more specific in diagnosing AC. Therefore, we concluded that when imaging is needed to confirm the diagnosis of AC, CT is the choice for patients who have atypical clinical signs and symptoms or unclear US findings, while US is the preferred initial modality for patients with typical manifestations of AC. Finally, we highly suggest conducting a multicenter study to assess the accuracy in different centers and age groups.
